# Targeting Lymphoma-associated Macrophage Expansion via CSF1R/JAK Inhibition is a Therapeutic Vulnerability in Peripheral T-cell Lymphomas

**DOI:** 10.1158/2767-9764.CRC-22-0336

**Published:** 2022-12-30

**Authors:** Xin Gao, Nermin Kady, Chenguang Wang, Suhaib Abdelrahman, Peter Gann, Maria Sverdlov, Ashley Wolfe, Noah Brown, John Reneau, Aaron M. Robida, Carlos Murga-Zamalloa, Ryan A. Wilcox

**Affiliations:** 1 Department of Internal Medicine, Division of Hematology and Oncology, University of Michigan, Ann Arbor, Michigan.; 2 Department of Pathology, University of Illinois Chicago, Chicago, Michigan.; 3 Department of Pathology, University of Michigan, Ann Arbor, Michigan.; 4 Department of Medicine, Division of Hematology, The Ohio State University Comprehensive Cancer Center, Columbus, Ohio.; 5 Life Sciences Institute, University of Michigan, Ann Arbor, Michigan.

## Abstract

The reciprocal relationship between malignant T cells and lymphoma-associated macrophages (LAM) within the tumor microenvironment (TME) is unique, as LAMs are well poised to provide ligands for antigen, costimulatory, and cytokine receptors that promote T-cell lymphoma growth. Conversely, malignant T cells promote the functional polarization and homeostatic survival of LAM. Therefore, we sought to determine the extent to which LAMs are a therapeutic vulnerability in these lymphomas, and to identify effective therapeutic strategies for their depletion. We utilized complementary genetically engineered mouse models and primary peripheral T-cell lymphoma (PTCL) specimens to quantify LAM expansion and proliferation. A high-throughput screen was performed to identify targeted agents that effectively deplete LAM within the context of PTCL. We observed that LAMs are dominant constituents of the TME in PTCL. Furthermore, their dominance was explained, at least in part, by their proliferation and expansion in response to PTCL-derived cytokines. Importantly, LAMs are a true dependency in these lymphomas, as their depletion significantly impaired PTCL progression. These findings were extrapolated to a large cohort of human PTCL specimens where LAM proliferation was observed. A high-throughput screen demonstrated that PTCL-derived cytokines led to relative resistance to CSF1R selective inhibitors, and culminated in the identification of dual CSF1R/JAK inhibition as a novel therapeutic strategy to deplete LAM in these aggressive lymphomas. Malignant T cells promote the expansion and proliferation of LAM, which are a *bone fide* dependency in these lymphomas, and are effectively depleted with a dual CSF1R/JAK inhibitor.

**Significance:**

LAMs are a therapeutic vulnerability, as their depletion impairs T-cell lymphoma disease progression. Pacritinib, a dual CSF1R/JAK inhibitor, effectively impaired LAM viability and expansion, prolonged survival in preclinical T-cell lymphoma models, and is currently being investigated as a novel therapeutic approach in these lymphomas.

## Introduction

Peripheral T-cell lymphomas (PTCL), derived from postthymic (“mature”) T cells, account for approximately 10%–15% of non–Hodgkin lymphomas (NHL) in North America (1). In stark contrast to the more common aggressive B-cell NHL, many of which are highly curable with modern immunochemotherapy regimens, the PTCL remains an area of unmet need. Despite recent advances (2), the most common PTCL remains “unclassifiable,” due to considerable genetic and molecular heterogeneity (3, 4). Intrinsic and acquired resistance to both conventional anthracycline-based chemotherapy and novel agents remains a persistent challenge (5, 6). Consequently, many patients afflicted with PTCL will succumb to their disease within a few years of diagnosis, and the PTCL increasingly accounts for a disproportionate number of NHL-related deaths. Among aggressive B-cell NHL, most notably diffuse large B-cell lymphoma (DLBCL), appreciation of both cell intrinsic (e.g., genetic and transcriptional) and extrinsic (e.g., microenvironmental) distinctions among DLBCL subtypes has significantly improved both DLBCL classification and risk stratification, but has also fostered the development of novel therapeutic strategies, including those exploiting the tumor microenvironment (TME; refs. 7, 8).

Despite their genetic and molecular heterogeneity and variable natural history, most PTCLs remain dependent upon their microenvironment, as constituents of the TME directly and indirectly promote the growth and survival of malignant T cells (9). Ligands and cytokines provided by constituents of the TME promote the growth and survival of malignant T cells upon binding their corresponding antigen, costimulatory, and cytokine receptors. Many of these receptors or their downstream signaling intermediates, while recurrently subjected to gain-of-function genetic alterations, remain dependent upon the provision of exogenous ligands by the TME (9, 10). Conversely, malignant T cells, via the cytokines they express, promote the variable recruitment, expansion, and functional polarization of constituents of the TME, thus creating a unique microenvironmental ecosystem. Prior to the advent of next-generation sequencing technology, conventional CD4^+^ T-cell subsets were largely defined by the cytokines they secrete, and their corresponding effects on the “microenvironment.” Not surprisingly then, PTCL ontology (or the “cell-of-origin”) is likely a significant determinant of microenvironmental, and subtype-specific, ecosystems. This principle is perhaps best illustrated by follicular helper T (T_FH_) cell-derived angioimmunoblastic T-cell lymphomas (AITL), as the production of T_FH_-associated cytokines in AITL drives the expansion of germinal center B cells and an expanded meshwork of follicular dendritic cells (FDC), creating a pathognomonic microenvironmental ecosystem (2).

In contrast to AITL, ecosystem-defining constituents of most other PTCL subtypes, and the extent to which they may be therapeutically exploited, are poorly understood. However, monocytes and macrophages (mono/mac) are abundant constituents of the TME in most PTCLs, and when present are associated with inferior outcomes (11–13), likely due to the repertoire of ligands they provide, and their role in suppressing host antitumor immunity (11, 14). The production of chemokines/cytokines that promote mono/mac recruitment and functional polarization may foster the development of a mono/mac-rich TME (4, 11, 15). If so, the extent to which malignant T cells promote mono/mac homeostatic survival and expansion, as has been described in other inflammatory states (16, 17), warrants scrutiny and has potentially significant therapeutic implications. Despite the expanding repertoire of therapies targeting (or exploiting) lymphoma-associated macrophages (LAM), their role in PTCL as a bona fide dependency generally, and the utility of LAM-depleting therapeutic strategies specifically, are incompletely understood and untested. Therefore, we sought to investigate the microenvironmental ecosystem and LAM dynamics in a genetically engineered PTCL model. Herein, we demonstrate that malignant T cells promote LAM expansion, and further demonstrate that LAMs are a dependency and therapeutic vulnerability within the PTCL ecosystem. Utilizing an unbiased, high-throughput screen, we have identified a multitargeted kinase (JAK/CSF1R) inhibitor (pacritinib) that depletes LAM and prolongs survival in PTCL-bearing mice, and is now being explored in an investigator-initiated clinical trial in PTCL (ClinicalTrials.gov Identifier: NCT04858256).

## Materials and Methods

### Mouse Models and *In Vivo* Experiments

Mouse studies were approved by the University Committee on Care and Use of Animals and performed in accordance with guidelines established by the Unit for Laboratory Animal Medicine at University of Michigan (Ann Arbor, MI). Mice were housed under specific-pathogen free conditions. P53 floxed, SNF5 floxed, and CD4-Cre mice were obtained from Jackson Laboratory and were crossed, and all F1 mice genotyped using tail DNA (Invitrogen Animal Tissue Direct PCR Kit). SNF5 mice were provided on a mixed background, but were backcrossed for at least 10 generations onto a B6 background. Mice generated during backcrossing were utilized for the experiments shown in Supplementary Fig. S1C, Supplementary Fig. S2A–S2D, and Supplementary Fig. S4A and S4B, whereas the remaining work shown here was obtained using mice fully backcrossed on a B6 background. Offspring with the desired genotype, including p53^fl/fl^ or ^+/+^, SNF5^fl/fl^, CD4-Cre^+^ (lymphoma-bearing mice) and p53^+/+^ or ^fl/+^, SNF5^fl/+^ or ^+/+^, CD-4-Cre^+^ (littermate controls), were utilized for these studies. Lymphoma development for the experiments described was determined by the development of easily palpable hepatosplenomegaly and/or bulky lymphadenopathy (>5 mm). For adoptive transfer experiment, 5 × 10^6^ bulk splenocytes obtained from lymphoma-bearing donor mice were retro-orbitally injected into 12–16 weeks old female C67BL/6 recipient mice. Treatment allocation was randomized, and all animals in given experiments were included for analysis. Mice were followed twice weekly for event-free survival (EFS), where an event is defined as the development of easily palpable hepatosplenomegaly and/or bulky lymphadenopathy and poor overall condition (e.g., “hunched and scruffy” appearance). Necropsy was performed at the time an event was observed, and spleen and liver weights determined. Single-cell suspensions were generated for further flow cytometric analysis. CD68-GFP reporter mice (strain #026827) and macrophage Fas-induced apoptosis (MaFIA) mice (strain #005070) were obtained from Jackson Laboratory, and were utilized as recipient mice. MaFIA mice in both control and experiment groups were treated with AP20187 (10 mg/kg) intranperitoneally for 5 consecutive days, and then once 3 days later, as described previously (18–20). Recipient mice were euthanized 4–5 days after the last dose of AP20187 and lymphoma burden examined. For pacritinib treatment, mice were provided either nutritionally complete control chow or chow supplemented with 0.3% pacritinib (Research Diets) *ad libitum* upon lymphoma engraftment. For the intraperitoneal tumor model, 1.5 × 10^7^ splenocytes from lymphoma-bearing mice were adoptively transferred into C57BL/6 mice by intraperitoneal injection. After spleens were palpable, mice were intraperitoneally injected with EdU (5-ethynyl-2′-deoxyuridine, 50 mg/kg) 16 hours before they were euthanized. Peritoneal lavage was performed with 5–10 mL PBS and flow cytometry performed.

### Antibodies, IHC, and Flow Cytometry

Commercially available (Supplementary Table S1), fluorochrome-conjugated antibodies (obtained from BD Biosciences, BioLegend, or eBioscience) were utilized for flow cytometry (CD11b, Ly6G, Ly6C, F4/80, CD115, CD193, SiglecF, CD117, CD16/32, CD34, Ki67). For intracellular (Ki67) staining, cells were fixed and permeabilized in Foxp3 Fix/Perm buffer (BioLegend) and then stained with appropriate antibodies. For EdU staining, cells were stained with CD11b and F4/80 antibodies first, then fix and stained with EdU permeabilized solution which contains EdU dye (EdU Cell proliferation kit, Base Click). Flow cytometry data were acquired using CyAn ADP Analyzer (Beckman Coulter) and the data were analyzed by FlowJo (BD Biosciences). For IHC, formalin-fixed, paraffin-embedded tissue sections were cut at 5 μm and rehydrated to water. Epitope retrieval was performed with proteinase K digestion at room temperature. Primary antibodies were applied after peroxidase blocking. Rabbit anti-rat biotinylated secondary antibody was applied and detected with streptavidin-horseradish peroxidase and DAB (3,3′-Diaminobenzidine) chromogen, as described previously (4). IHC for eosinophil major basic protein (MBP) was performed as described previously (21), and the antibody kindly provided by Dr. James J. Lee and Dr. Nancy A. Lee. Slides were viewed using an Olympus BX51 microscope and pictures taken with an Olympus DP71 camera. Olympus BSW with DP Controller software was used for image acquisition and storage.

### Cell Culture and Drug Screen

H9 (HTB-176) and SUDHL1 (CRL-2955) cells were purchased from ATCC. MyLa CD4^+^ (95051032) and MyLa CD8^+^ (95051033) were purchased from Millipore Sigma. Immortalized mouse bone marrow–derived macrophage cell line was provided by Dr. Hedeki Hara (Keio University School of Medicine). Karpas 299, SR-786, and Mac-1 cells were kindly provided by Dr. Megan Lim (University of Pennsylvania, Philadelphia, PA). T8ML-1 cells were kindly provided by Drs. Fujiwara and Yasukawa, and cultured as described previously (14). MyLa cells were kindly provided by Dr. Robert Gniadecki (University of Alberta, Edmonton, Canada). Unless indicated otherwise, cells were cultured in RPMI1640 supplemented with 10% FBS. For the generation of cell-free conditioned media (CFCM), cells were resuspended in fresh media and plated at 1 × 10^6^/mL density and cultured for 24 hours. For generation of CFCM from primary malignant T cells, malignant T cells were sorted using CD3 or CD4 microbeads (Miltenyi Biotec) from peripheral blood obtained from patients with Sezary syndrome with significant (>85% of peripheral blood lymphocytes) leukemic involvement. To maintain viability, primary malignant T cells were cultured at 1 × 10^6^/mL density with anti-CD3/CD28 Dynabeads (Thermo Fisher Scientific #11132D), as described previously (14). Supernatants were harvested and centrifuged at 2,800 rpm using a Centrifuge 5810 R (Eppendorf) for 5 minutes to remove any cells. After centrifugation, supernatants were filtered using low-protein binding 5 μm filter (Millipore), and supernatants used immediately, or stored for up to 2 weeks at −20°C. Cytokines (CSF1, IL4, IL10, IL13, IFNγ) were quantified by sandwich ELISA (RayBiotech) according to the manufacturer's instructions. All cell lines were *Mycoplasma* free and independently authenticated by short tandem repeat profiling, performed by ATCC, and immunophenotyping. CD14 microbeads (Miltenyi Biotec) were used to isolate monocytes from healthy donors (HD). Monocytes were cultured with different CFCM (50% volume for volume in RPMI1640 supplemented with 10% FBS), in RPMI1640 supplemented with 10% FBS alone, or in media supplemented with the cytokines (CSF1, IL4, IL13) indicated. Cytokines were low endotoxin, premium grade, and were utilized at 20 ng/mL (Miltenyi Biotec). For viability studies, 5 × 10^4^ cells/well were plated in triplicate in 96-well plates, and monocyte viability determined 48–72 hours later by RealTime-Glo (Promega), and the data normalized to monocyte viability at time 0. For the high-throughput screen, monocytes were plates at a density of 5,000 cells per well in 50 μL of medium into Griener 781080 white cell culture 384-well plates. At time of cell plating, a baseline viability measurement was performed (as detailed below) for normalization. Monocytes were grown in either primary patient-derived CFCM (50%) or CSF1 (20 ng/mL). Stock compounds were solvated in DMSO at 2 mmol/L. Compound delivery to assay plates was performed using a MosquitoX1 (TTP Labtech). Compounds were tested in triplicate at a final concentration of 2 μmol/L (using columns 3 to 22 of the assay plate). Negative controls medium only plus matching 0.1% DMSO were included in columns 1 and 2. Control conditioned medium wells (with 0.1% DMSO) were also included for reference in columns 23 and 24 of each assay plate. Following compound addition, cells were cultured for 48 hours at 5% CO_2_ at 37°C. Cell viability was measured using CellTiter-Glo luminescent kit (catalog no. G7571) from Promega as directed using a PHERAstar instrument from BMG Labtech.

### Statistics Analysis

Data analyses were performed in GraphPad Prism 8.0 package. Comparisons between groups were evaluated using two-tailed Student *t* test or one-way ANOVA and *P* values <0.05 considered statistically significant. EFS was summarized with Kaplan–Meier method, and comparisons made with log-rank test.

Please see the Supplementary Materials and Methods for additional information about RNA sequencing (RNA-seq) and multispectral imaging.

### Data and Materials Availability

All data are available in the main text or the Supplementary Materials and Methods. The genetically engineered mouse (GEM) models generated may be provided upon request, in compliance with local and institutional guidelines, and upon execution of a materials transfer agreement. Sequencing data have been publicly deposited (accession PRJNA839400).

## Results

### LAMs are Dominant Constituents of the Microenvironmental Ecosystem and a Dependency in PTCL

Members of the SWI/SNF family of chromatin remodeling proteins are recurrently mutated or deleted in mature T-cell lymphomas (22–27), and conditional deletion of *SNF5* in mice leads to the development of a spontaneous PTCL with complete penetrance (28). We performed bulk RNA-seq in sorted CD3^+^ T cells obtained from littermate control (*SNF5*^+/+^ or ^fl/+^, CD4-Cre^+^), and both young (<4 months of age) and older (>4 months) *SNF5*^fl/fl^, CD4-Cre^+^ mice. Consistent with prior observations, the emergence of clonal T cells, accompanied by the development of clinically significant lymphadenopathy and/or hepatosplenomegaly, is exclusively observed in older (>4 months) mice (28). While we have performed bulk RNA-seq studies in sorted CD3^+^ T cells, we exploited the observation that cellular and/or RNA contamination is largely unavoidable in bulk RNA-seq datasets (29–31), and hypothesized that the cellular and/or RNA contamination observed provides a transcriptional “footprint” of the microenvironmental ecosystem. Therefore, gene expression signatures reflecting relevant lymphocyte-, myeloid-, and nonhematopoietic-derived constituents of the TME were generated and applied to our RNA-seq dataset (Supplementary Data S1). Disease evolution upon SNF5 deletion and the emergence of a bona fide PTCL was associated with significant transcriptional changes within the microenvironmental ecosystem. Despite a significant loss of B-cell and natural killer (NK)-cell signatures (Supplementary Fig. S1A and S1B), a macrophage signature was retained.

**FIGURE 1:**
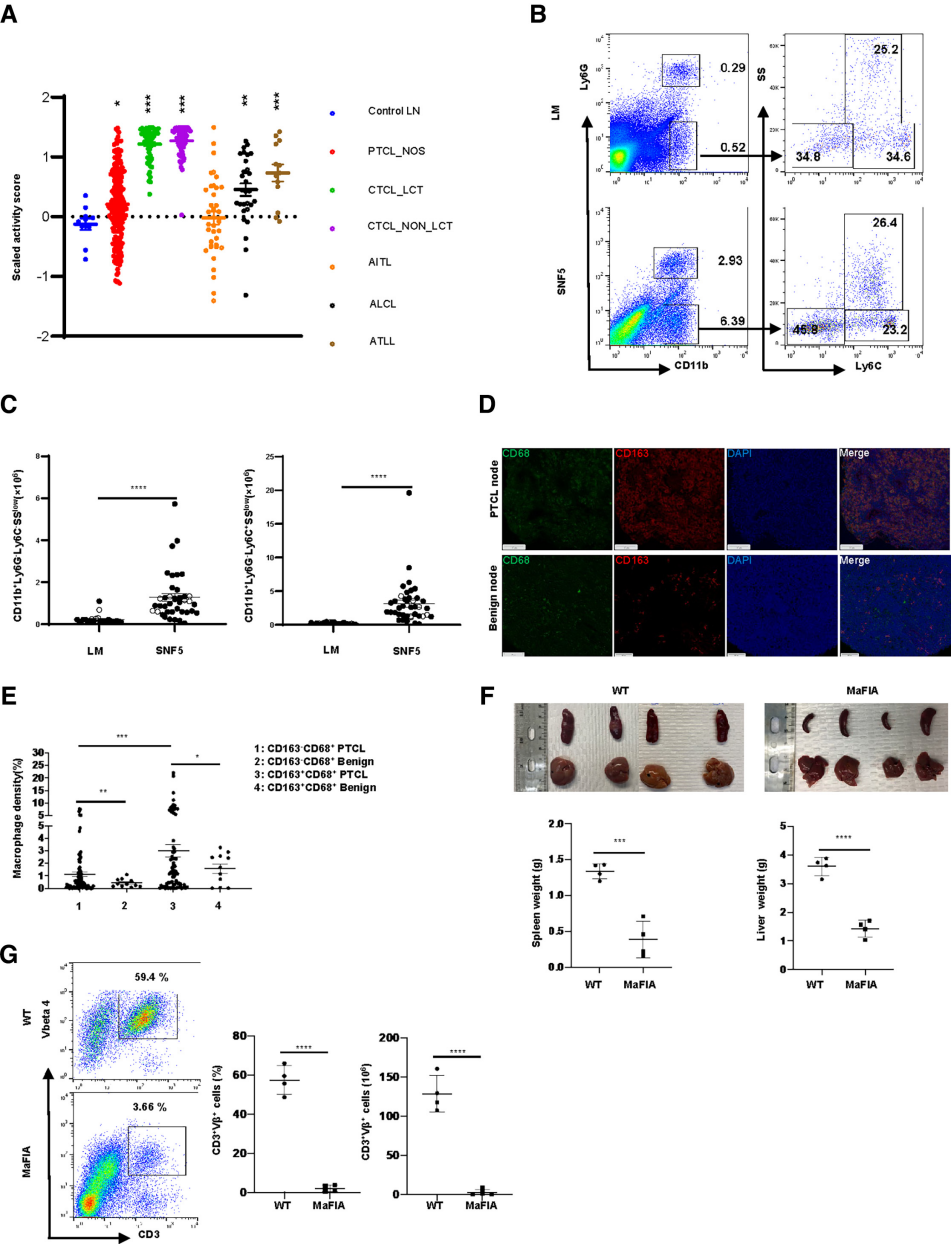
PTCLs are dependent upon mono/mac within the TME and promote their expansion. **A,** A gene expression signature, comprised of macrophage-related transcripts, was examined across the PTCL subtypes indicated (peripheral T-cell lymphoma, not otherwise specified, PTCL, NOS; cutaneous T-cell lymphoma with large cell transformation, CTCL_LCT; CTCL without large cell transformation, CTCL_non_LCT; angioimmunoblastic T-cell lymphoma, AITL; anaplastic large cell lymphoma, ALCL; adult T-cell leukemia/lymphoma, ATLL) and benign lymph nodes (control LN). **B** and **C,** Splenic mono/mac were quantified by flow cytometry in lymphoma-bearing SNF5^fl/fl^, p53^+/+^, CD4-Cre (closed circle, *n* = 30), SNF5^fl/fl^, p53^fl/fl^, CD4-Cre (opened circle, *n* = 4) and littermate control mice (LM, *n* = 24). A representative example of classical monocytes (CD11b^+^Ly6G^−^Ly6C^hi^SS^low^) and nonclassical mono/mac (CD11b^+^Ly6G^−^Ly6C^low^) is shown in **B**, and summarized results are shown in **C**. **D** and **E,** Multispectral imaging of PTCL, NOS lymph nodes (*n* = 78), and benign lymph nodes (*n* = 11) was performed to determine CD68^+^ macrophage densities, stratified by CD163 expression. On average, 28,025 total cells were examined per biopsy. A representative image is shown in **D** and summarized in **E**. **F** and **G,** Splenocytes from lymphoma-bearing SNF5^fl/fl^, p53^+/+^, CD4-Cre were adoptively transferred into syngeneic control or MaFIA recipient mice (*n* = 4/group). Upon lymphoma engraftment, determined by the development of palpable splenomegaly, mice in both groups were treated with AP20187 and euthanized 10 days later. Disease burden, determined by spleen/liver weights (**F**) and quantification of clonal T cells in splenocytes using a lymphoma-specific TCR-Vβ antibody (**G**) was determined (*, *P* < 0.05; **, *P* < 0.01; ***, *P* < 0.001; ****, *P* < 0.0001).

In various human PTCL and cutaneous T-cell lymphoma (CTCL) subtypes, with the exception of AITL (where B cells and FDC play a prominent role in the TME), a significant enrichment in macrophage-related transcripts was observed (Fig. 1A). To validate these findings in our GEM model, spleens from both littermate control and lymphoma-bearing mice were examined by IHC, and a significant infiltrate of F4/80^+^ macrophages was observed in lymphoma-bearing mice, while NK cells were largely excluded (Supplementary Figs. S1C–S1E, S2A, and S2B). A significant expansion of both classical monocytes (CD11b^+^Ly6G^−^Ly6C^hi^) and nonclassical mono/mac (CD11b^+^Ly6G^−^Ly6C^lo^) was also observed (Fig. 1B and C). To further bolster the clinical relevance of this model, we crossed SNF5 and p53 floxed mice, as p53 deletions are highly recurrent in the most aggressive and chemorefractory PTCL (26). Conditional knockout of p53 in this model accelerated PTCL development, as observed in other lymphoma models (32). The median time to PTCL development was 156 days in p53+ PTCL and 65 days in p53-deficient PTCL (*P* < 0.0001). Regardless of p53 status, a similarly expanded population of mono/mac was observed in these mice (Fig. 1C). The mono/mac expansion observed was attributed, at least in part, to a corresponding increase in bone marrow common monocyte progenitors (33); and a generalized increase in myelopoiesis, including an expanded pool of mature neutrophils and eosinophils (Supplementary Figs. S2C–S2F and S3).

We quantified the density of LAM in human PTCL using multispectral imaging (*n* = 78) and compared the results with benign/reactive (*n* = 11) lymph nodes. Macrophages were defined as CD3^−^CD8^−^CD20^−^CD68^+^ cells, and were further stratified by CD163 expression, as PTCL-derived cytokines have been previously shown to polarize LAM, inducing CD163 expression (4). Compared with benign lymph nodes, and consistent with the extent of mono/mac expansion observed in lymphoma-bearing mice, an approximately 5-fold and 2-fold increase in CD163^−^ and CD163^+^ LAM densities, respectively, were observed in PTCL lymph nodes (Fig. 1D and E).

Malignant T cells emerging in SNF5^fl/f^, CD4-Cre^+^ mice, despite their proliferative capacity *in vivo*, undergo rapid spontaneous apoptosis when cultured *ex vivo* (Supplementary Fig. S4A and S4B), suggesting that the provision of extrinsic growth and survival signals by constituents of the TME support lymphoma growth (11). Therefore, we evaluated the role of LAM on the viability of malignant T cells by culturing splenocytes from lymphoma-bearing mice alone, or in the presence of syngeneic macrophages (Supplementary Fig. S4C and S4D). Consistent with previous observations in human T-cell lymphomas (11), macrophages significantly increased the viability of malignant T cells *ex vivo*. However, to examine the extent to which LAMs are a true PTCL dependency *in vivo*, splenocytes from lymphoma-bearing mice were adoptively transferred into either B6 or MaFIA recipient mice. MaFIA mice express the Fas intracellular domain fused with a mutant human FK506-binding protein under control of the *Csf1r* promoter. Administration of the dimerization drug AP20187 leads to dimerization of the transgene and Fas-induced apoptosis of monocytes and tissue-resident macrophages (18). Upon PTCL engraftment, mice were treated with AP20187, which depleted LAM, as anticipated (Supplementary Fig. S5), but also led to a significant reduction in disease burden (Fig. 1F and G). Consistent with our *ex vivo* data, and prior studies (11), these data demonstrate that LAMs are a bona fide dependency in PTCL. Consequently, LAM depletion is a rational therapeutic strategy.

### LAM Expansion and Proliferation in PTCL

**FIGURE 2:**
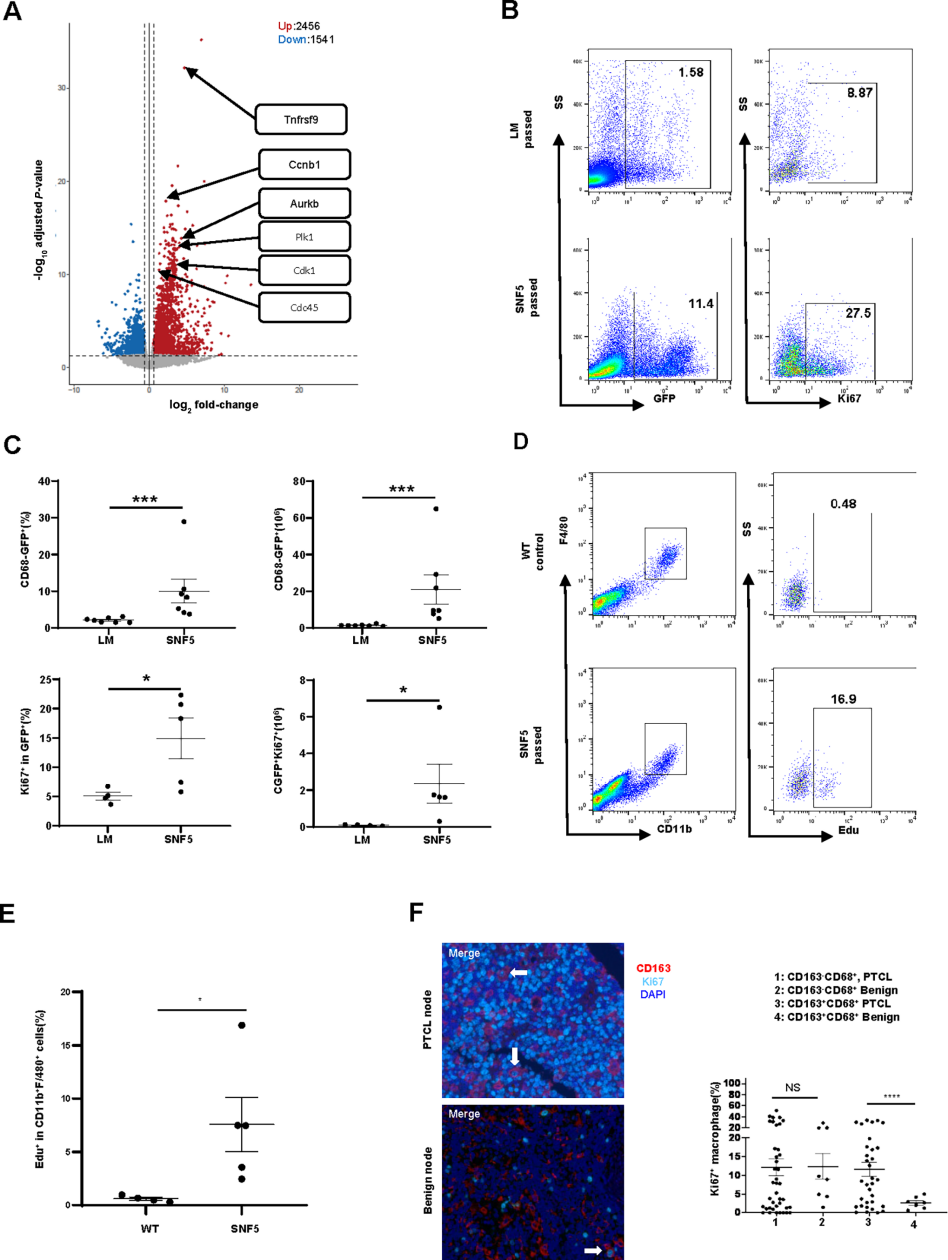
PTCL transcriptionally reprogram and induce the proliferation of mono/mac within the TME. **A,** Splenocytes from lymphoma-bearing SNF5^fl/fl^, CD4-Cre (SNF5, *n* = 5) or littermate control mice (LM, *n* = 3) were adoptively transfer into CD68-GFP reporter mice. After PTCL engraftment, determined by the development of palpable hepatosplenomegaly, both control and lymphoma-bearing mice were euthanized and splenocytes harvested. GFP^+^ cells were sorted and RNA-seq performed. A volcano plot comparing gene expression between LAM obtained from control and lymphoma-bearing mice is shown. **B** and **C,** Splenocytes from lymphoma-bearing SNF5^fl/fl^, CD4-Cre (SNF5, *n* = 7) or littermate control mice (LM, *n* = 7) were adoptively transferred into CD68-GFP reporter mice. Upon engraftment, GFP^+^ LAM and Ki67 expression were examined by flow cytometry. A representative example is shown in **B** and summarized in **C**. **D** and **E,** Splenocytes from lymphoma-bearing SNF5^fl/fl^, CD4-Cre mice (*n* = 5) or LM controls (*n* = 4) were injected intraperitoneally into C57BL/6 recipient mice. After tumor engrafted, recipient mice were injected (intraperitoneally) with EdU, and mice euthanized 16 hours later, and peritoneal cells harvested. EdU incorporation in CD11b^+^F4/80^+^ peritoneal macrophages was examined. A representative example is shown in **D** and summarized in **E**. **F,** Multispectral imaging of PTCL, NOS lymph nodes (*n* = 78), and benign lymph nodes (*n* = 11) was performed using the macrophage markers CD68 and CD163, and proliferation examined by Ki67. The percentage of Ki67^+^ macrophages, stratified by CD163 expression, was determined. Representative images are shown, and proliferating (Ki67^+^) macrophages highlighted (white arrow). The data are summarized at right (*, *P* < 0.05; **, *P* < 0.01; ***, *P* < 0.001; ****, *P* < 0.0001).

Malignant T cells produce cytokines that functionally polarize LAM (4) and stimulate mono/mac proliferation in inflammatory conditions (16, 17). Therefore, we sought to examine the extent to which LAM proliferation may additionally contribute to their expansion. To do so, we employed CD68-GFP reporter mice, as mono/mac from these mice express GFP. Splenocytes obtained from littermate control or lymphoma-bearing donor mice were adoptively transferred into CD68-GFP recipients. Upon engraftment, GFP^+^ cells were sorted, and RNA-seq performed. In contrast to GFP^+^ mono/mac obtained from control recipients, those obtained from lymphoma-bearing recipients were transcriptionally reprogrammed and significantly enriched for transcripts associated with cell proliferation (Fig. 2A; Supplementary Fig. S6; Supplementary Data S2). Consistent with these findings, GFP^+^ mono/mac significantly expanded in lymphoma-bearing mice and expressed Ki67 (Fig. 2B and C), which identifies proliferating macrophages (16). As LAM expansion and proliferation may be context dependent, and to examine an alternative population of tissue-resident macrophages, the proliferation of peritoneal LAM in lymphoma-bearing mice was examined by EdU incorporation. In comparison with peritoneal macrophages obtained from control mice, EdU incorporation (Fig. 2D and E) was detectable in peritoneal LAM, further supporting the view that T-cell lymphomas promote cell-cycle entry and proliferation of LAM.

We next examined LAM proliferation by Ki67 in human PTCL using multispectral imaging. Proliferating CD163^+^CD68^+^ LAM were observed in PTCL lymph nodes, and approximately four times more abundant than those observed in benign lymph nodes (Fig. 2F). A significant difference in the proliferation of CD163^−^CD68^+^ LAM was not observed, suggesting that PTCL-derived cytokines, previously associated with the induction of CD163 expression (4), may promote LAM proliferation in human PTCL.

### Dual CSF1R/JAK Inhibitors Impair LAM Expansion and Proliferation

**FIGURE 3:**
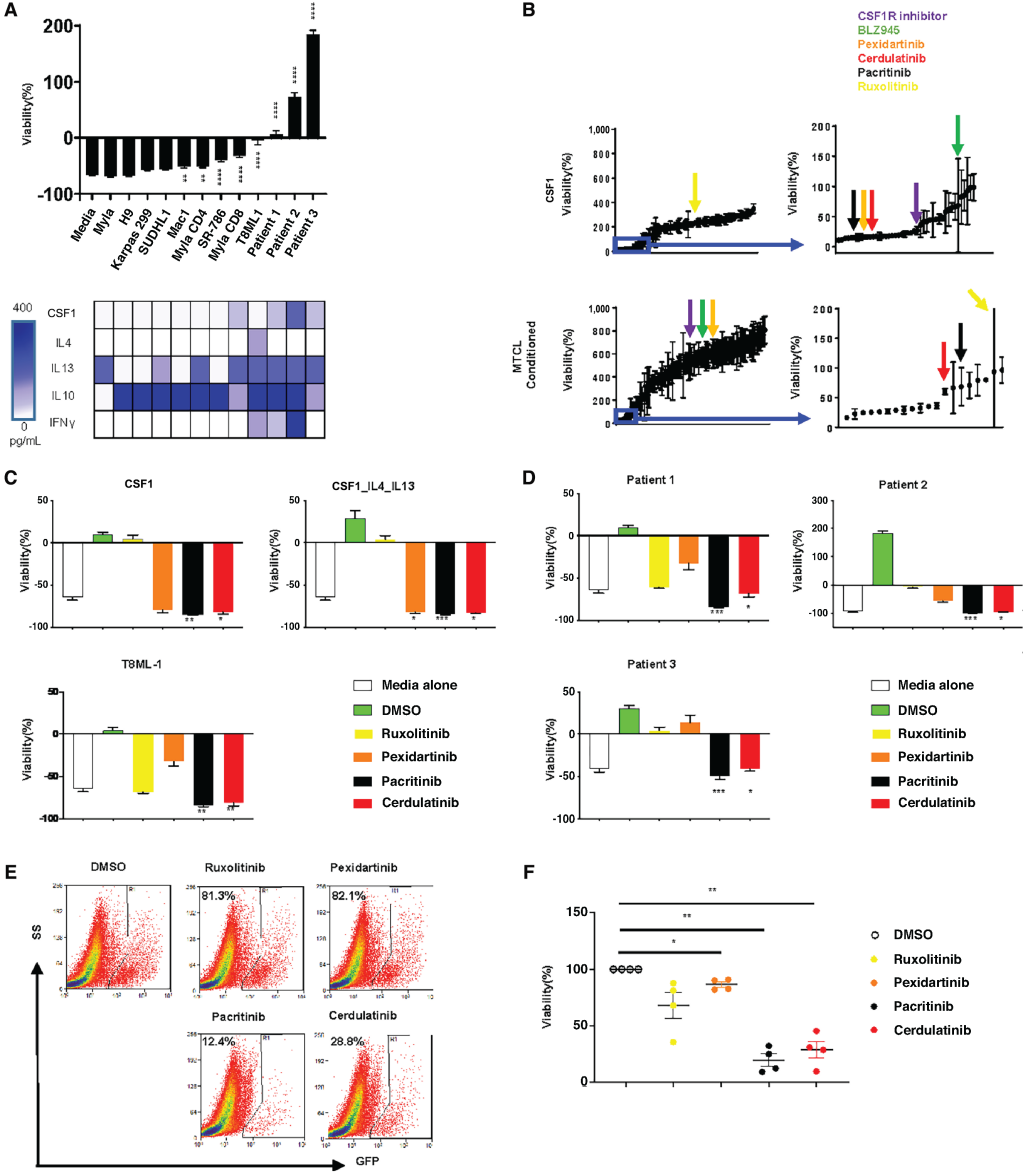
Mono/mac expansion is impaired by dual CSF1R/JAK inhibitors. **A,** Human CD14^+^ monocytes obtained from HDs were cultured in media alone, or in media supplemented with (50%) CFCM obtained from the human PTCL cell lines or primary specimens, as indicated. Monocyte viability was determined 72 hours later in comparison with media alone, and upon normalization to monocyte viability at time 0. CSF1, IL4, IL13, IL10, and IFNγ were quantified in the CFCM by sandwich ELISA, and that data summarized below. **B,** Normal donor monocytes were cultured with rhCSF1 (top) or with CFCM (bottom) obtained from a primary TCL specimen. Viability was determined 72 hours later (and normalized to viability at time 0) in triplicate using a 384-well format. Experiments were performed using a library of 191 targeted agents (indicated on *x*-axis). Cytocidal agents (normalized viability <100%) are shown at right. Selected agents, including CSF1R inhibitors (“CSF1R inhibitor,” BLZ945, and pexidartinib), ruxolitinib, pacritinib, and cerdulatinib are indicated with the arrows shown. Similarly designed and independent experiments were performed using recombinant cytokines or T8ML-1 CFCM (**C**) and primary patient CFCM (**D**) in the presence of ruxolitinib (1 μmol/L), pexidartinib (2 μmol/L), pacritinib (1 μmol/L), cerdulatinib (1 μmol/L), or vehicle control (DMSO). **E** and **F,** Splenocytes from lymphoma-bearing SNF5^fl/fl^, CD4-Cre mice (*n* = 4) were adoptively transferred into CD68-GFP reporter recipient mice. Upon lymphoma engraftment, splenocytes from reporter mice were cultured in the presence of ruxolitinib (1 μmol/L), pexidartinib (2 μmol/L), pacritinib (1 μmol/L), cerdulatinib (1 μmol/L) or vehicle control (DMSO) and GFP^+^ cells quantified by flow cytometry 48 hours later. The percentage of GFP^+^ cells in treated groups was normalized in a pairwise fashion to the DMSO group. A representative example is shown in **E** and the normalized viability data summarized in **F** (*, *P* < 0.05; **, *P* < 0.01; ***, *P* < 0.001; ****, *P* < 0.0001).

**FIGURE 4:**
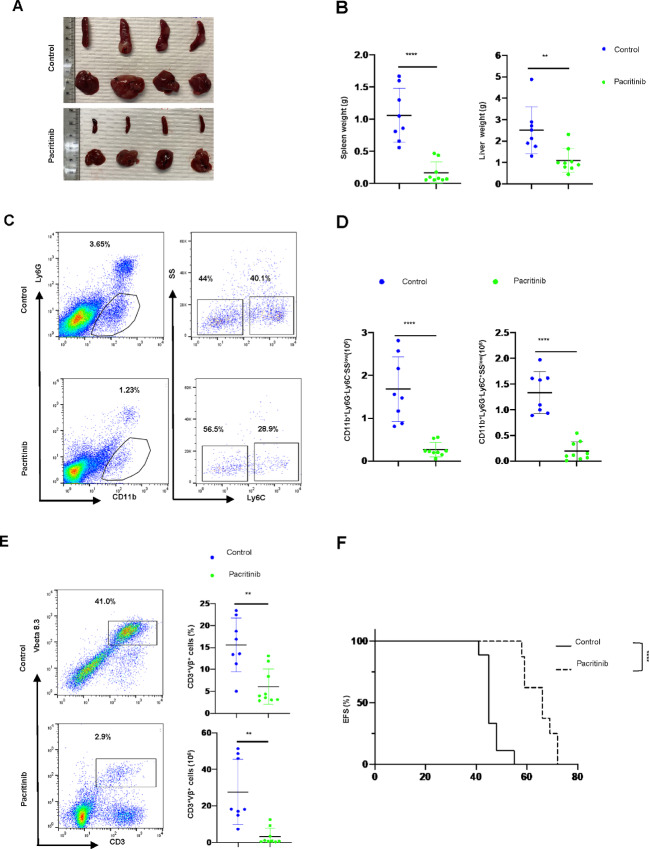
The targeted CSF1R/JAK inhibitor pacritinib depletes PTCL-associated macrophages *in vivo* and prolongs survival in lymphoma-bearing mice. **A–E,** Splenocytes from lymphoma-bearing SNF5 ^fl/fl^, p53^+/+^ or ^fl/fl^, CD4-Cre^+^ mice were adoptively transferred into C57BL/6J recipient mice (*n* = 4–5 recipients/experimental group in two biological replicates) and treated with either control or pacritinib-containing (0.3%) chow upon lymphoma engraftment. Mice were euthanized when mice in the control group were moribund. Explanted spleens and livers were weighed (**A**), and organ weights summarized (**B**). Mono/mac densities in splenocytes were determined by flow cytometry (**C** and **D**), as before. Disease burden (determined by the expression of clonal and lymphoma-specific TCR-Vβ) was quantified by flow cytometry. A representative example is shown and the data summarized (**E**). **F,** Similarly designed experiments were conducted (*n* = 4–5 recipients/experimental group in two biological replicates, independent from those utilized in **A**–**D**) and recipient mice treated with control or pacritinib-containing chow, and mice followed for EFS (*, *P* < 0.05; **, *P* < 0.01; ***, *P* < 0.001; ****, *P* < 0.0001).

To further explore that possibility, we generated human monocyte-derived macrophages, using CD14^+^ monocytes, obtained from HDs, cultured with CFCM obtained from human mature T-cell lymphoma (MTCL) cell lines and primary specimens. Monocyte viability was determined 72 hours later. Compared with culture media alone, and upon normalization to monocyte viability at time 0, we observed that CFCM obtained from a subset of MTCL lines and primary specimens promoted monocyte viability and/or expansion (Fig. 3A). Furthermore, we observed that CSF1, a homeostatic cytokine essential for the viability of monocytes and tissue-resident macrophages, and inflammatory cytokines (e.g., IL4/IL13) associated with mono/mac proliferation, were detectable in the conditioned media obtained from those cell lines that efficiently drove monocyte viability and/or expansion (Fig. 3A). We next performed a high-throughput screen with a library of 191 agents (Supplementary Data S3) to identify targeted agents that inhibit LAM expansion. As anticipated, CSF1R tyrosine kinase inhibitors (TKI) were identified in our screen using HD monocytes cultured in the presence of rhCSF1. Surprisingly, and in contrast, monocytes cultured in the presence of CFCM obtained from primary malignant T cells were only marginally impaired by CSF1R inhibitors, whereas the JAK inhibitors ruxolitinib, cerdulatinib, and pacritinib significantly impaired monocyte viability and expansion (Fig. 3B; Supplementary Fig. S7). Unlike ruxolitinib or pexidartinib, which selectively inhibit JAKs and CSF1R, respectively, cerdulatinib and pacritinib inhibit both JAKs and CSF1R at submicromolar concentrations (Supplementary Table S2). Pacritinib, for example, in addition to inhibiting CSF1R, selectively inhibits JAK2, JAK3, and TYK2 at low nanomolar concentrations (Supplementary Table S2), and may thus be described as a “JAK/CSF1R” inhibitor. In subsequent confirmatory experiments, both cerdulatinib and pacritinib significantly impaired monocyte viability and expansion in the presence of T-cell lymphoma-derived cytokines present within CFCM (Fig. 3C and D). In contrast to pexidartinib and ruxolitinib, which are relatively selective for CSF1R and JAK1/JAK2, respectively, both cerdulatinib (34) and pacritinib (35) are dual JAK and CSF1R inhibitors, suggesting that CSF1, while necessary, is unlikely sufficient to drive LAM expansion. Given the therapeutic implications, we extended these findings to our GEM mouse model using CD68-GFP reporter mice, and observed that both cerdulatinib and pacritinib significantly depleted LAM *ex vivo* in this model (Fig. 3E and F). To examine the extent to which pacritinib may deplete LAM and prolong survival *in vivo*, lymphoma-bearing mice were treated with pacritinib-containing chow. A significant reduction in spleen and liver weights (Fig. 4A and B), macrophage depletion (Fig. 4C and D), decreased disease burden (Fig. 4E), and prolonged survival (Fig. 4F) were observed in pacritinib-treated mice. It is notable that the T-cell lymphomas in this model are chemorefractory, as a negligible survival benefit is observed upon administration of conventional chemotherapeutic agents (36), whereas pacritinib prolonged median EFS by approximately 20 days in this aggressive and chemorefractory model.

## Discussion

Despite the cell-autonomous effects of SNF5 and/or p53 loss, malignant T cells in this model, much like primary malignant T cells obtained from patients (11, 37), remained dependent upon the TME, as spontaneous apoptosis was preferentially observed in clonal T cells upon *ex vivo* culture. Furthermore, malignant T cells shape their local ecosystem by regulating the recruitment, expansion, and functional polarization of its constituents. Herein, we demonstrate that LAMs are important constituents of the TME, and their sheer abundance is explained, at least in part, by their proliferation. In addition, LAMs are a bona fide dependency *in vivo*, as their depletion significantly reduced disease burden. Our findings are consistent with those recently described by Cortes and colleagues using a Vav1-Myo1f transgenic model (15). In this model, emergence of a GATA-3+ PTCL that transcriptionally resembles a subset of human PTCL and produces Th2-associated cytokines (4), including those previously implicated in driving macrophage cell-cycle entry and proliferation (16, 17), were associated with macrophage expansion. Furthermore, clodronate-based LAM depletion decreased lymphoma burden in this model. Consequently, therapeutic strategies to effectively deplete LAM warrant further study in well-designed clinical trials.

LAM depletion (35, 38), or at least their functional attenuation (4, 39), has emerged as an attractive therapeutic strategy across many hematologic malignancies and solid tumors. The accumulation of myeloid-derived cells at inflammatory sites has been historically attributed to the recruitment of mature myeloid cells or their progenitors, as tissue-resident macrophages, while functionally malleable, were thought to be quiescent. This view has been challenged, as the *in situ* proliferation of mono/mac has been observed in multiple inflammatory states (16, 17, 40) and in response to multiple cytokines (17, 41), including those that are abundant in PTCL subtypes characterized by dense LAM infiltrates (4, 13, 15). Our observations that LAM are transcriptionally reprogrammed, enriched for cell cycle–related and proliferation-related transcripts, express Ki67, and incorporate EdU demonstrate that their expansion is explained, at least in part, by cell-cycle entry and proliferation, and not merely a generalized increase in myelopoiesis or mono/mac recruitment. To the best of our knowledge, our findings using multispectral imaging in PTCL biopsy specimens provide the first direct evidence of LAM proliferation *in situ* in any lymphoma. This finding has significant clinical implications, as current therapeutic strategies to deplete tumor-associated macrophages are TKI- or antibody-based and selectively directed at CSF1R (38). However, while CSF1 production was observed by human MTCL cell lines and primary patient samples, and almost certainly promotes the homeostatic survival of LAM, the production of other inflammatory cytokines, including those previously implicated in driving macrophage proliferation, likely promote LAM proliferation. This hypothesis is further supported by our unbiased, high-throughput screen demonstrating that, at least within a T-cell lymphoma context, LAMs are relatively “resistant” to selective CSF1R antagonists, and is compatible with similar findings in other model systems (42, 43). However, in contrast to these prior studies, we have further demonstrated that two clinically available JAK/CSF1R inhibitors effectively deplete LAM. While CSF1R is an attractive therapeutic target in PTCL (44), and JAK inhibitors are highly effective agents in disorders associated with macrophage activation (45), our observations suggest that dual JAK/CSF1R inhibition may be required for optimal LAM depletion in PTCL. This hypothesis is being further tested in an ongoing clinical trial with pacritinib in relapsed or refractory T-cell lymphomas (ClinicalTrials.gov Identifier: NCT04858256).

Of course, our study also has significant limitations, as a depletional strategy targeting LAM fails to account for alternative therapeutic approaches, a number of which may be antagonistic with the approach adopted here. As the TME in many PTCL's is permissive for LAM, alternative therapeutic approaches which seek to exploit LAM as effector cells, upon CD47/SIRPα blockade (39), for example, are certainly rational and effective in a minority of patients in early-phase clinical studies. Alternatively, targeted agents, including JAK and PI3Kγ inhibitors, may effectively “transform” LAM by influencing their polarization state (4, 46–48). Future studies investigating strategies to deplete, exploit, or transform LAM, and the development of novel combinatorial strategies, including the incorporation of immunotherapeutic approaches, are certainly justified.

In summary, we demonstrate that malignant T cells promote a robust expansion and proliferation of LAM, which are a true dependency in PTCL. These findings have significant therapeutic implications, as dual JAK/CSF1R inhibitors effectively impaired the expansion and proliferation of LAM, depletion of which decreased disease burden and prolonged survival in a GEM model.

## Supplementary Material

Fig. S1 The microenvironmental ecosystem in lymphoma-bearing mice

Fig. S2 PTCL associated neutrophil and eosinophil expansion.

Fig. S3 PTCL induced myelopoiesis.

Fig. S4 Spontaneous apoptosis of malignant T cells ex vivo

Fig. S5 Macrophage depletion in MaFIA mice.

Fig. S6 Fig. S6: GSEA in LAM

Fig. S7 Identification of dual CSF-1R/JAK inhibitors in high-throughput screen

Table TS1 Antibodies used

Table TS2 JAK inhibitor kinase profile

Data File DS1 TME gene sets

Data File DS2 RNA-seq demonstrates transcriptional reprogramming of LAM

Data File DS3 Agents used in high-throughput screen

## References

[1] Vose J , Armitage J, Weisenburger D; International T-Cell Lymphoma Project. International peripheral T-cell and natural killer/T-cell lymphoma study: pathology findings and clinical outcomes. J Clin Oncol 2008;26:4124–30. 18626005 10.1200/JCO.2008.16.4558

[2] Iqbal J , Wilcox R, Naushad H, Rohr J, Heavican TB, Wang C, . Genomic signatures in T-cell lymphoma: how can these improve precision in diagnosis and inform prognosis? Blood Rev 2016;30:89–100. 26319391 10.1016/j.blre.2015.08.003

[3] Iqbal J , Wright G, Wang C, Rosenwald A, Gascoyne RD, Weisenburger DD, . Gene expression signatures delineate biological and prognostic subgroups in peripheral T-cell lymphoma. Blood 2014;123:2915–23. 24632715 10.1182/blood-2013-11-536359 PMC4014836

[4] Wang T , Feldman AL, Wada DA, Lu Y, Polk A, Briski R, . GATA-3 expression identifies a high-risk subset of PTCL, NOS with distinct molecular and clinical features. Blood 2014;123:3007–15. 24497534 10.1182/blood-2013-12-544809 PMC4014843

[5] Mak V , Hamm J, Chhanabhai M, Shenkier T, Klasa R, Sehn LH, . Survival of patients with peripheral T-cell lymphoma after first relapse or progression: spectrum of disease and rare long-term survivors. J Clin Oncol 2013;31:1970–6. 23610113 10.1200/JCO.2012.44.7524

[6] Zhang JY , Briski R, Devata S, Kaminski MS, Phillips TJ, Mayer TL, . Survival following salvage therapy for primary refractory peripheral T-cell lymphomas (PTCL). Am J Hematol 2018;93:394–400. 29194714 10.1002/ajh.24992 PMC5803354

[7] Schmitz R , Wright GW, Huang DW, Johnson CA, Phelan JD, Wang JQ, . Genetics and pathogenesis of diffuse large B-cell lymphoma. N Engl J Med 2018;378:1396–407. 29641966 10.1056/NEJMoa1801445 PMC6010183

[8] Kotlov N , Bagaev A, Revuelta MV, Phillip JM, Cacciapuoti MT, Antysheva Z, . Clinical and biological subtypes of B-cell lymphoma revealed by microenvironmental signatures. Cancer Discov 2021;11:1468–89. 33541860 10.1158/2159-8290.CD-20-0839 PMC8178179

[9] Wilcox RA . A three-signal model of T-cell lymphoma pathogenesis. Am J Hematol 2016;91:113–22. 26408334 10.1002/ajh.24203 PMC4715594

[10] Fiore D , Cappelli LV, Broccoli A, Zinzani PL, Chan WC, Inghirami G. Peripheral T cell lymphomas: from the bench to the clinic. Nat Rev Cancer 2020;20:323–42. 32249838 10.1038/s41568-020-0247-0

[11] Wilcox RA , Wada DA, Ziesmer SC, Elsawa SF, Comfere NI, Dietz AB, . Monocytes promote tumor cell survival in T-cell lymphoproliferative disorders and are impaired in their ability to differentiate into mature dendritic cells. Blood 2009;114:2936–44. 19671921 10.1182/blood-2009-05-220111 PMC2756204

[12] Zhang W , Wang L, Zhou D, Cui Q, Zhao D, Wu Y. Expression of tumor-associated macrophages and vascular endothelial growth factor correlates with poor prognosis of peripheral T-cell lymphoma, not otherwise specified. Leuk Lymphoma 2011;52:46–52. 10.3109/10428194.2010.529204 21077742

[13] Zhang W , Wang Z, Luo Y, Zhong D, Luo Y, Zhou D. GATA3 expression correlates with poor prognosis and tumor-associated macrophage infiltration in peripheral T cell lymphoma. Oncotarget 2016;7:65284–94. 27589565 10.18632/oncotarget.11673 PMC5323155

[14] Wang T , Lu Y, Polk A, Chowdhury P, Zamalloa CM, Fujiwara H, . T-cell receptor signaling activates an ITK/NF-kappaB/GATA-3 axis in T-cell lymphomas facilitating resistance to chemotherapy. Clin Cancer Res 2017;23:2506–15. 27780854 10.1158/1078-0432.CCR-16-1996 PMC5405012

[15] Cortes JR , Filip I, Albero R, Patiño-Galindo JA, Quinn SA, Lin W-HW, . Oncogenic Vav1-Myo1f induces therapeutically targetable macrophage-rich tumor microenvironment in peripheral T cell lymphoma. Cell Rep 2022;39:110695. 35443168 10.1016/j.celrep.2022.110695 PMC9059228

[16] Jenkins SJ , Ruckerl D, Cook PC, Jones LH, Finkelman FD, van Rooijen N, . Local macrophage proliferation, rather than recruitment from the blood, is a signature of TH2 inflammation. Science 2011;332:1284–8. 21566158 10.1126/science.1204351 PMC3128495

[17] Jenkins SJ , Ruckerl D, Thomas GD, Hewitson JP, Duncan S, Brombacher F, . IL-4 directly signals tissue-resident macrophages to proliferate beyond homeostatic levels controlled by CSF-1. J Exp Med 2013;210:2477–91. 24101381 10.1084/jem.20121999 PMC3804948

[18] Burnett SH , Kershen EJ, Zhang J, Zeng L, Straley SC, Kaplan AM, . Conditional macrophage ablation in transgenic mice expressing a Fas-based suicide gene. J Leukoc Biol 2004;75:612–23. 14726498 10.1189/jlb.0903442

[19] Soki FN , Cho SW, Kim YW, Jones JD, Park SI, Koh AJ, . Bone marrow macrophages support prostate cancer growth in bone. Oncotarget 2015;6:35782–96. 26459393 10.18632/oncotarget.6042 PMC4742141

[20] Clifford AB , Elnaggar AM, Robison RA, O'Neill K. Investigating the role of macrophages in tumor formation using a MaFIA mouse model. Oncol Rep 2013;30:890–6. 23722325 10.3892/or.2013.2508

[21] Lee JJ , Dimina D, Macias MP, Ochkur SI, McGarry MP, O'Neill KR, . Defining a link with asthma in mice congenitally deficient in eosinophils. Science 2004;305:1773–6. 15375267 10.1126/science.1099472

[22] Kiel MJ , Sahasrabuddhe AA, Rolland DCM, Velusamy T, Chung F, Schaller M, . Genomic analyses reveal recurrent mutations in epigenetic modifiers and the JAK-STAT pathway in Sezary syndrome. Nat Commun 2015;6:8470. 26415585 10.1038/ncomms9470 PMC4598843

[23] Wang L , Ni X, Covington KR, Yang BY, Shiu J, Zhang X, . Genomic profiling of Sezary syndrome identifies alterations of key T cell signaling and differentiation genes. Nat Genet 2015;47:1426–34. 26551670 10.1038/ng.3444 PMC4829974

[24] da Silva Almeida AC , Abate F, Khiabanian H, Martinez-Escala E, Guitart J, Tensen CP, . The mutational landscape of cutaneous T cell lymphoma and Sezary syndrome. Nat Genet 2015;47:1465–70. 26551667 10.1038/ng.3442 PMC4878831

[25] Choi J , Goh G, Walradt T, Hong BS, Bunick CG, Chen K, . Genomic landscape of cutaneous T cell lymphoma. Nat Genet 2015;47:1011–9. 26192916 10.1038/ng.3356 PMC4552614

[26] Heavican TB , Bouska A, Yu J, Lone W, Amador C, Gong Q, . Genetic drivers of oncogenic pathways in molecular subgroups of peripheral T-cell lymphoma. Blood 2019;133:1664–76. 30782609 10.1182/blood-2018-09-872549 PMC6460420

[27] Watatani Y , Sato Y, Miyoshi H, Sakamoto K, Nishida K, Gion Y, . Molecular heterogeneity in peripheral T-cell lymphoma, not otherwise specified revealed by comprehensive genetic profiling. Leukemia 2019;33:2867–83. 31092896 10.1038/s41375-019-0473-1

[28] Wang X , Werneck MBF, Wilson BG, Kim HJ, Kluk MJ, Thom CS, . TCR-dependent transformation of mature memory phenotype T cells in mice. J Clin Invest 2011;121:3834–45. 21926465 10.1172/JCI37210 PMC3195451

[29] Nieuwenhuis TO , Yang SY, Verma RX, Pillalamarri V, Arking DE, Rosenberg AZ, . Consistent RNA sequencing contamination in GTEx and other data sets. Nat Commun 2020;11:1933. 32321923 10.1038/s41467-020-15821-9 PMC7176728

[30] Millard SM , Heng O, Opperman KS, Sehgal A, Irvine KM, Kaur S, . Fragmentation of tissue-resident macrophages during isolation confounds analysis of single-cell preparations from mouse hematopoietic tissues. Cell Rep 2021;37:110058. 34818538 10.1016/j.celrep.2021.110058

[31] Sicherman J , Newton DF, Pavlidis P, Sibille E, Tripathy SJ. Estimating and correcting for off-target cellular contamination in brain cell type specific RNA-seq data. Front Mol Neurosci 2021;14:637143. 33746712 10.3389/fnmol.2021.637143 PMC7966716

[32] Liu J , Chen G, Feng L, Zhang W, Pelicano H, Wang F, . Loss of p53 and altered miR15-a/16-1 MCL-1 pathway in CLL: insights from TCL1-Tg:p53(-/-) mouse model and primary human leukemia cells. Leukemia 2014;28:118–28. 23608884 10.1038/leu.2013.125 PMC3806892

[33] Hettinger J , Richards DM, Hansson J, Barra MM, Joschko AC, Krijgsveld J, . Origin of monocytes and macrophages in a committed progenitor. Nat Immunol 2013;14:821–30. 23812096 10.1038/ni.2638

[34] Coffey G , Betz A, DeGuzman F, Pak Y, Inagaki M, Baker DC, . The novel kinase inhibitor PRT062070 (Cerdulatinib) demonstrates efficacy in models of autoimmunity and B-cell cancer. J Pharmacol Exp Ther 2014;351:538–48. 25253883 10.1124/jpet.114.218164

[35] Polk A , Lu Y, Wang T, Seymour E, Bailey NG, Singer JW, . Colony-stimulating factor-1 receptor is required for nurse-like cell survival in chronic lymphocytic leukemia. Clin Cancer Res 2016;22:6118–28. 27334834 10.1158/1078-0432.CCR-15-3099 PMC5161678

[36] Geng X , Wang C, Gao X, Chowdhury P, Weiss J, Villegas JA, . GATA-3 is a proto-oncogene in T-cell lymphoproliferative neoplasms. Blood Cancer J 2022;12:149. 36329027 10.1038/s41408-022-00745-y PMC9633835

[37] Lyu A , Triplett TA, Nam SH, Hu Z, Arasappan D, Godfrey WH, . Tumor-associated myeloid cells provide critical support for T-ALL. Blood 2020;136:1837–50. 32845007 10.1182/blood.2020007145

[38] Hume DA , MacDonald KPA. Therapeutic applications of macrophage colony-stimulating factor-1 (CSF-1) and antagonists of CSF-1 receptor (CSF-1R) signaling. Blood 2012;119:1810–20. 22186992 10.1182/blood-2011-09-379214

[39] Jain S , Van Scoyk A, Morgan EA, Matthews A, Stevenson K, Newton G, . Targeted inhibition of CD47-SIRPα requires Fc-FcγR interactions to maximize activity in T-cell lymphomas. Blood 2019;134:1430–40. 31383641 10.1182/blood.2019001744 PMC6839960

[40] Campbell SM , Knipper JA, Ruckerl D, Finlay CM, Logan N, Minutti CM, . Myeloid cell recruitment versus local proliferation differentiates susceptibility from resistance to filarial infection. Elife 2018;7:e30947. 29299998 10.7554/eLife.30947 PMC5754202

[41] Jackson-Jones LH , Rückerl D, Svedberg F, Duncan S, Maizels RM, Sutherland TE, . IL-33 delivery induces serous cavity macrophage proliferation independent of interleukin-4 receptor alpha. Eur J Immunol 2016;46:2311–21. 27592711 10.1002/eji.201646442 PMC5082546

[42] Pradel LP , Ooi CH, Romagnoli S, Cannarile MA, Sade H, Rüttinger D, . Macrophage susceptibility to emactuzumab (RG7155) treatment. Mol Cancer Ther 2016;15:3077–86. 27582524 10.1158/1535-7163.MCT-16-0157

[43] Pyonteck SM , Akkari L, Schuhmacher AJ, Bowman RL, Sevenich L, Quail DF, . CSF-1R inhibition alters macrophage polarization and blocks glioma progression. Nat Med 2013;19:1264–72. 24056773 10.1038/nm.3337 PMC3840724

[44] Murga-Zamalloa C , Rolland DCM, Polk A, Wolfe A, Dewar H, Chowdhury P, . Colony-stimulating factor 1 receptor (CSF1R) activates AKT/mTOR signaling and promotes T-cell lymphoma viability. Clin Cancer Res 2020;26:690–703. 31636099 10.1158/1078-0432.CCR-19-1486 PMC7002219

[45] Ahmed A , Merrill SA, Alsawah F, Bockenstedt P, Campagnaro E, Devata S, . Ruxolitinib in adult patients with secondary haemophagocytic lymphohistiocytosis: an open-label, single-centre, pilot trial. Lancet Haematol 2019;6:e630–7. 31537486 10.1016/S2352-3026(19)30156-5 PMC8054981

[46] Kaneda MM , Messer KS, Ralainirina N, Li H, Leem CJ, Gorjestani S, . PI3Kγ is a molecular switch that controls immune suppression. Nature 2016;539:437–42. 27642729 10.1038/nature19834 PMC5479689

[47] Horwitz SM , Koch R, Porcu P, Oki Y, Moskowitz A, Perez M, . Activity of the PI3K-δ,γ inhibitor duvelisib in a phase 1 trial and preclinical models of T-cell lymphoma. Blood 2018;131:888–98. 29233821 10.1182/blood-2017-08-802470 PMC5824337

[48] Moskowitz AJ , Ghione P, Jacobsen E, Ruan J, Schatz JH, Noor S, . A phase 2 biomarker-driven study of ruxolitinib demonstrates effectiveness of JAK/STAT targeting in T-cell lymphomas. Blood 2021;138:2828–37. 34653242 10.1182/blood.2021013379 PMC8718625

