# Supplementary material for: Targeting Lymphoma-associated Macrophage Expansion via CSF1R/JAK Inhibition is a Therapeutic Vulnerability in Peripheral T-cell Lymphomas
Source: Cancer Res Commun. 2022 Dec 30;2(12):1727–37. doi: 10.1158/2767-9764.CRC-22-0336 (PMC10035520; doi:10.1158/2767-9764.CRC-22-0336)
Supplement: Fig. S1 — The microenvironmental ecosystem in lymphoma-bearing mice [file crc-22-0336-s01.docx]

**
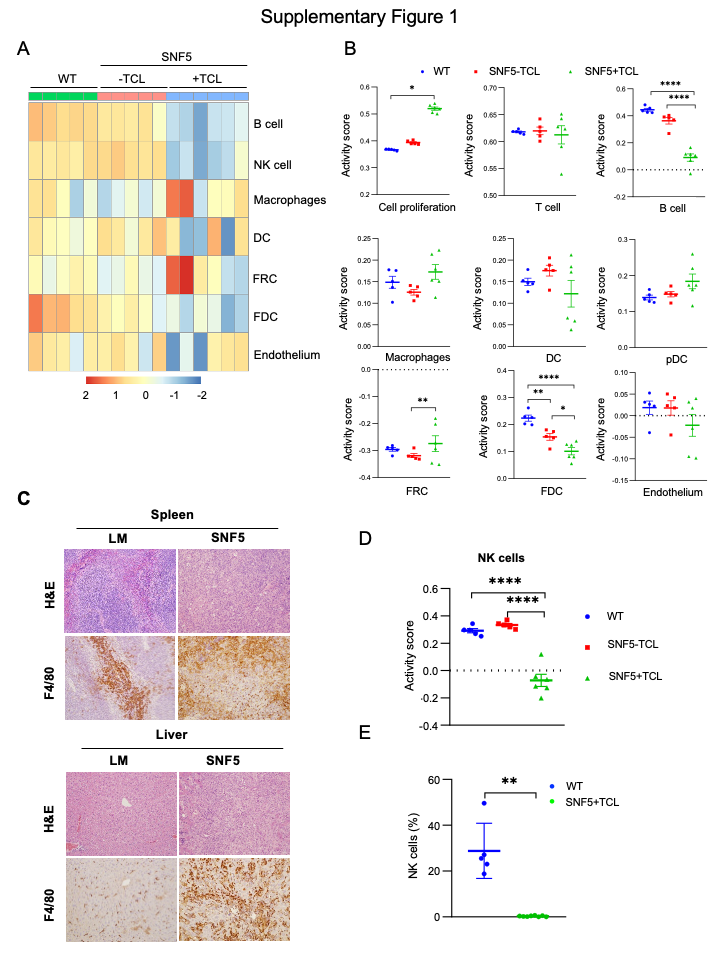
**

**Supplementary Figure 1.** **The microenvironmental ecosystem in lymphoma-bearing SNF^fl/fl^, CD4-Cre mice.** CD3+ T cells were sorted from littermate control (WT, n=5), young (<4 months of age) SNF5^fl/fl^, CD4-cre mice without PTCL (SNF5 -TCL, n=5), and from older SNF5^fl/fl^, CD4-cre mice with PTCL (SNF5 +TCL, n=6) and subjected to bulk RNA-seq. (A, B) Gene enrichment scores were generated using B-cell, NK cell, macrophage, dendritic cell (DC), fibroblastic reticular cell (FRC), follicular dendritic cell (FDC), and endothelial-related transcripts and a supervised analysis performed. Summary data is shown (A), and individual activity scores for each TME-related signatured summarized (B). (C) Lymphoma-associated macrophages were examined in spleens and livers obtained from littermate control mice (LM) or SNF5^fl/fl^, CD4-cre, lymphoma-bearing mice (SNF5) by F4/80 immunohistochemistry. A representative example (n>10) is shown. (D) NK cell activity score was compared between WT, SNF5-TCL and SNF5+TCL mice, and relative exclusion of NK cells in lymphoma-bearing mice observed. (E) This was validated using splenocytes from independent WT and SNF5+TCL mice. The frequency of Lin(CD3, CD19, Ly6G)^-^NK1.1^+^CD49b^+^ NK cells is summarized. (* P<0.05, ** P<0.01, *** P<0.001, **** P<0.0001)
