# Supplementary material for: Targeting Lymphoma-associated Macrophage Expansion via CSF1R/JAK Inhibition is a Therapeutic Vulnerability in Peripheral T-cell Lymphomas
Source: Cancer Res Commun. 2022 Dec 30;2(12):1727–37. doi: 10.1158/2767-9764.CRC-22-0336 (PMC10035520; doi:10.1158/2767-9764.CRC-22-0336)
Supplement: Fig. S2 — PTCL associated neutrophil and eosinophil expansion. [file crc-22-0336-s02.docx]

**
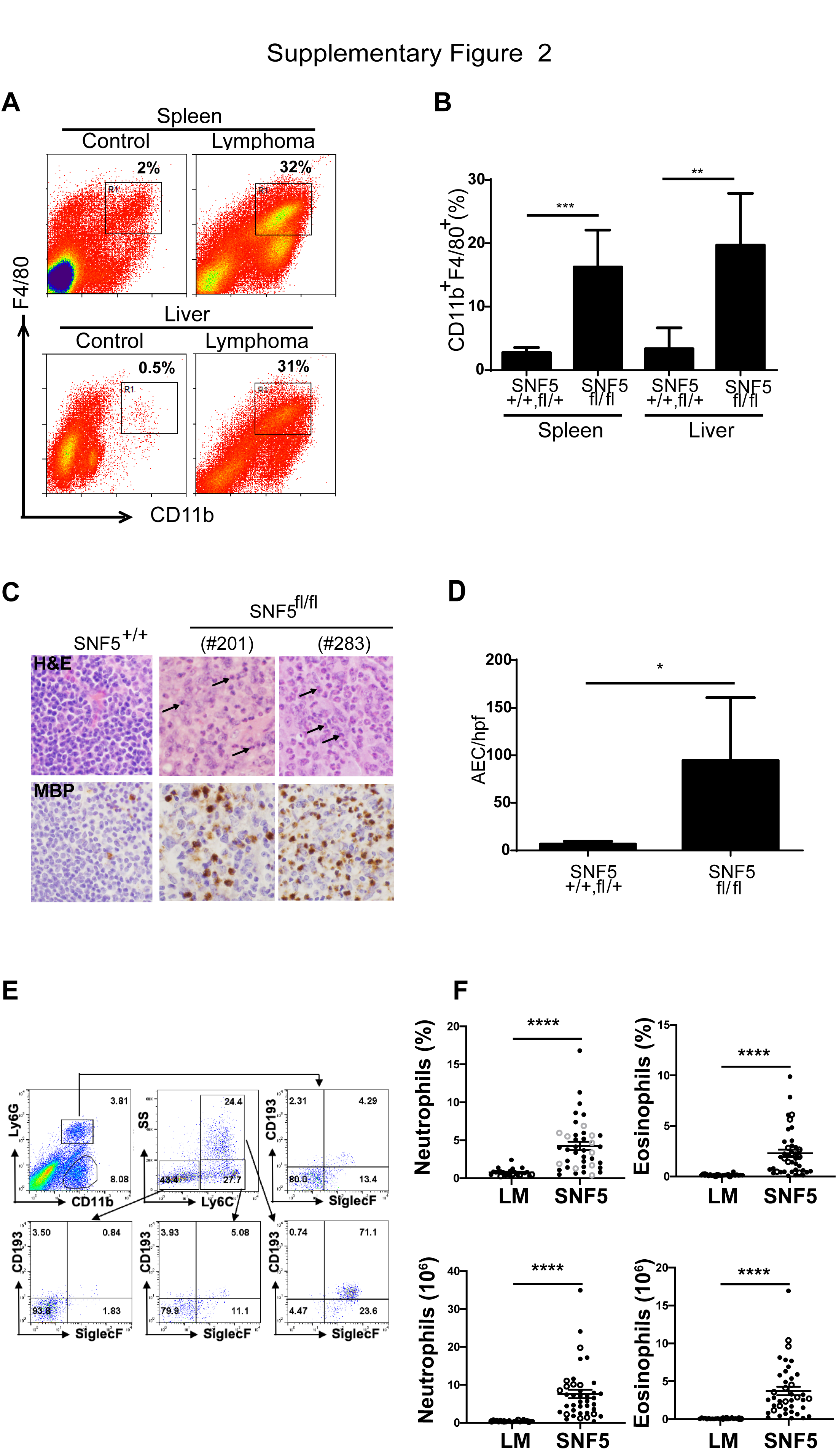
**

**Supplementary Figure 2.** **PTCL development is associated with the expansion of lymphoma-associated neutrophils and eosinophils.** (A, B) CD11b^+^F4/80^+^ lymphoma-associated macrophages were examined by flow cytometry in the spleen (n=8) and liver (n=4) from lymphoma-bearing SNF5^fl/fl^, CD4-Cre^+^ (SNF5) or littermate controls (LM, n=10) mice, as indicated. A representative example is shown in (A), and summarized in (B). (C, D) Lymphoma-associated eosinophils (LAE) are appreciated on H&E staining of spleens obtained from lymphoma-bearing mice (arrows in C) and identified by immunohistochemistry for major basic protein (MBP), as indicated. Representative examples are shown in (C), and the density of LAE per high-power field (400x) in littermate controls (n=4) and lymphoma-bearing mice (n=4) summarized in (D). (E, F) Splenocytes from lymphoma-bearing SNF5^fl/fl^, CD4-Cre^+^ (closed circle, n=30), SNF5 ^fl/fl^, p53^fl/fl,^ CD4-Cre^+^ (opened circle, n=4) and littermate control mice (LM, n=24) were identified with pan-myeloid panel by flow cytometry. A representative example from lymphoma-bearing SNF5^fl/fl^, CD4-Cre^+^ mice is shown to identify the lymphoma-associated eosinophils (Ly6G^-^CD11b^+^SS^high^CD193^+^SiglecF^+^) in (E). The expansion of neutrophils (Ly6G^+^CD11b^+^) and eosinophils (Ly6G^-^CD11b^+^SS^high^) are summarized in (F). (* P<0.05, ** P<0.01, *** P<0.001, **** P<0.0001)
