# Supplementary material for: Targeting Lymphoma-associated Macrophage Expansion via CSF1R/JAK Inhibition is a Therapeutic Vulnerability in Peripheral T-cell Lymphomas
Source: Cancer Res Commun. 2022 Dec 30;2(12):1727–37. doi: 10.1158/2767-9764.CRC-22-0336 (PMC10035520; doi:10.1158/2767-9764.CRC-22-0336)
Supplement: Fig. S3 — PTCL induced myelopoiesis. [file crc-22-0336-s03.docx]

**
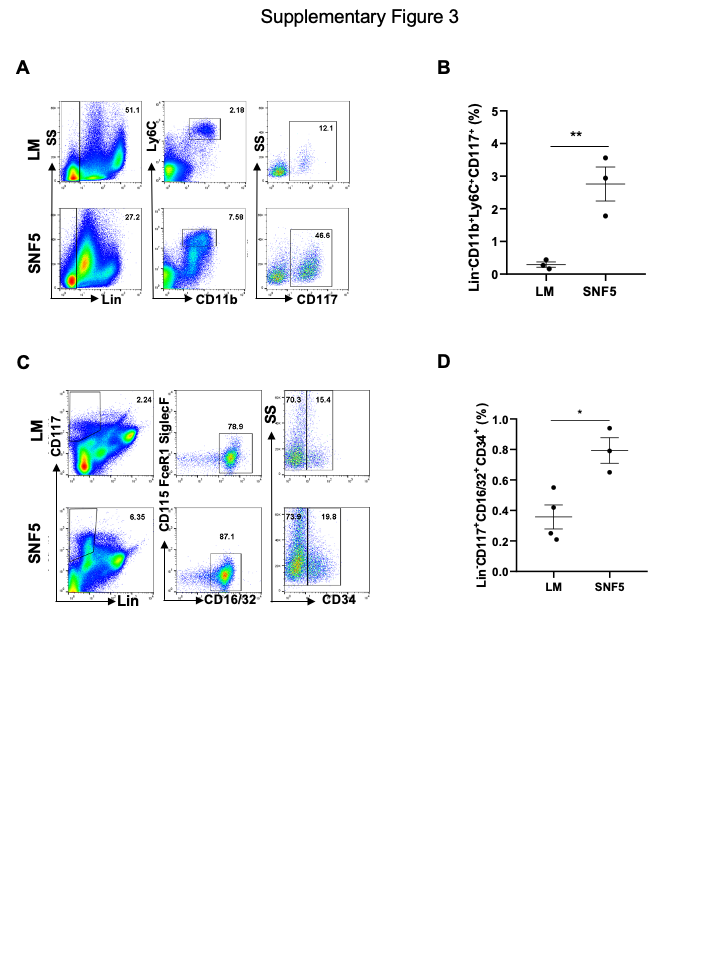
**

**Supplementary Figure 3.** **PTCL induces a generalized increase in myelopoiesis.** (A, B) Common monocyte progenitors (Lin^-^CD11b^+^Ly6C^+^CD117^+^) were examined in bone marrow cells from lymphoma-bearing SNF5^fl/f^, CD4-Cre (SNF5, n=3) and littermate control (LM, n=3) mice. A representative example is shown in (A), and summarized in (B). (C, D) Neutrophil progenitors (Lin^-^CD117^+^CD16/32^+^CD115-FceR1^-^SiglecF^-^CD34^+^) in bone marrow cells from SNF5 (n=3) and LM (n=4) mice were similarly examined. A representative example is shown in (C), and summarized in (D). (* P<0.05, ** P<0.01, *** P<0.001, **** P<0.0001)
