# Supplementary material for: Targeting Lymphoma-associated Macrophage Expansion via CSF1R/JAK Inhibition is a Therapeutic Vulnerability in Peripheral T-cell Lymphomas
Source: Cancer Res Commun. 2022 Dec 30;2(12):1727–37. doi: 10.1158/2767-9764.CRC-22-0336 (PMC10035520; doi:10.1158/2767-9764.CRC-22-0336)
Supplement: Fig. S4 — Spontaneous apoptosis of malignant T cells ex vivo [file crc-22-0336-s04.docx]

**
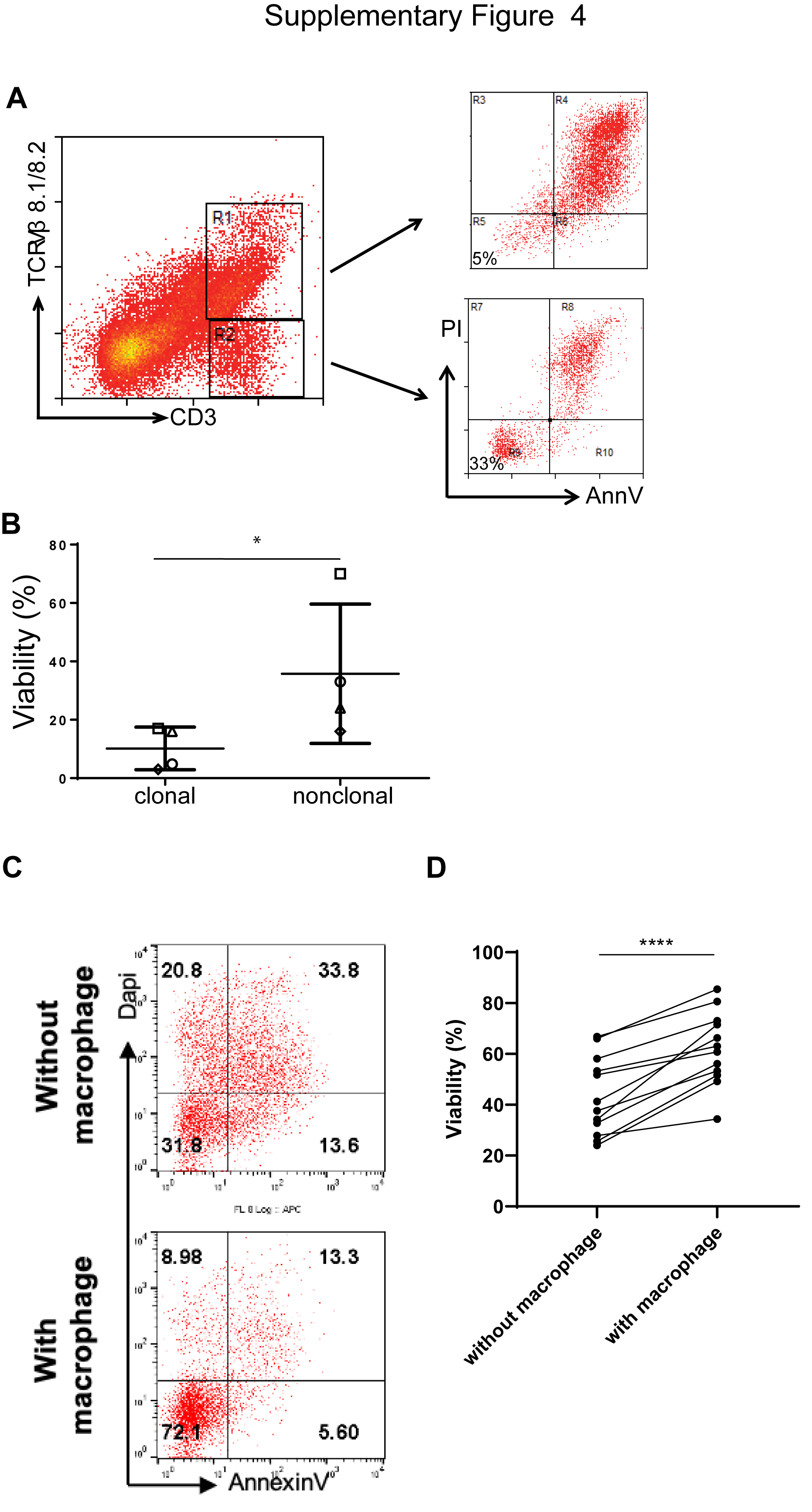
**

**Supplementary Figure 4.** **Malignant T cells from SNF5^fl/f^, CD4-Cre lymphoma-bearing mice undergo rapid spontaneous apoptosis *ex vivo* and partially rescued by co-cultured with macrophage.** (A, B) Splenocytes (5x10^6^/well of 24-well plate) from lymphoma-bearing SNF5^fl/f^, CD4-Cre mice were cultured *ex vivo* for 72 hours and the viability of clonal (Vβ^+^) and non-clonal (Vβ^-^) T cells examined by annexin V and propidium iodide (PI) staining by flow cytometry. A representative example is shown in (A) and summarized in (B). (C, D) Splenocytes from lymphoma-bearing SNF5^fl/f^, CD4-Cre mice were similarly cultured in the presence or absence of a syngeneic, bone-marrow derived macrophage cell line for 48hrs, and the viability of clonal T cells determined by annexin V and DAPI staining. A representative example is shown in (C) and summarized in (D). (* P<0.05, ** P<0.01, *** P<0.001, **** P<0.0001)
