# Supplementary material for: Targeting Lymphoma-associated Macrophage Expansion via CSF1R/JAK Inhibition is a Therapeutic Vulnerability in Peripheral T-cell Lymphomas
Source: Cancer Res Commun. 2022 Dec 30;2(12):1727–37. doi: 10.1158/2767-9764.CRC-22-0336 (PMC10035520; doi:10.1158/2767-9764.CRC-22-0336)
Supplement: Fig. S5 — Macrophage depletion in MaFIA mice. [file crc-22-0336-s05.docx]

**
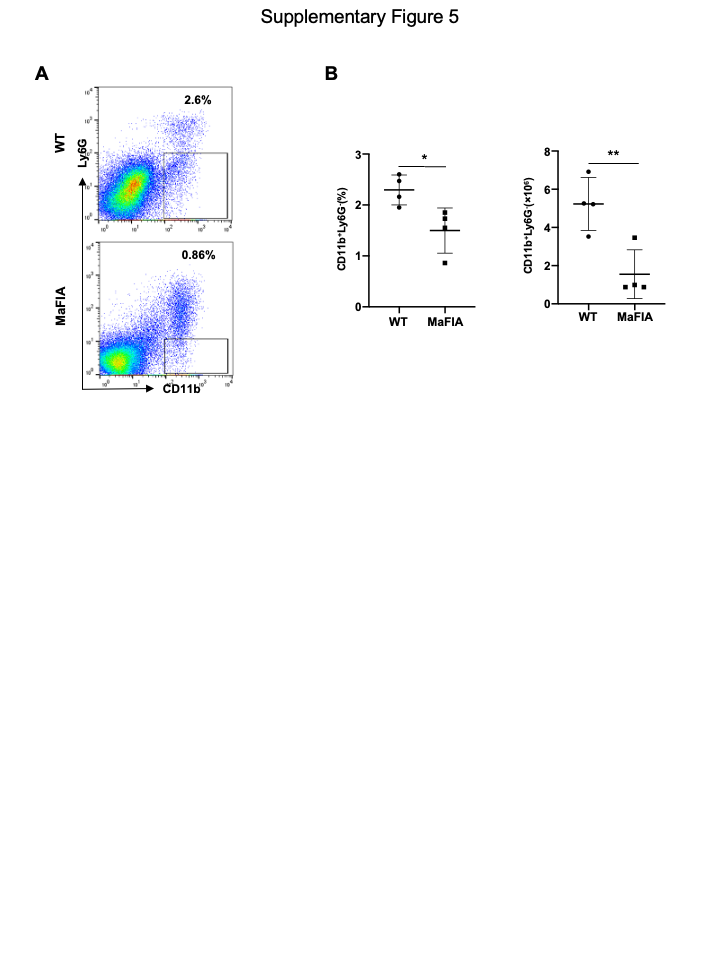
**

**Supplementary Figure 5.** **Administration of AP20187 in MaFIA mice deplete lymphoma-associated macrophages.** (A, B) Splenocytes from lymphoma-bearing SNF5^fl/fl^, CD4-Cre were adoptively transferred into control B6 (WT) or MaFIA recipient mice (n=4/group). Upon lymphoma engraftment, determined by the development of palpable splenomegaly, mice in both groups were treated with AP20187. All mice were euthanized after treatment (day 10), and Ly6G^-^CD11b^+^ mono/mac density in splenocytes was determined by flow cytometry. A representative example is shown in (A), and summarized in (B). (* P<0.05, ** P<0.01, *** P<0.001, **** P<0.0001)
