# Supplementary material for: Targeting Lymphoma-associated Macrophage Expansion via CSF1R/JAK Inhibition is a Therapeutic Vulnerability in Peripheral T-cell Lymphomas
Source: Cancer Res Commun. 2022 Dec 30;2(12):1727–37. doi: 10.1158/2767-9764.CRC-22-0336 (PMC10035520; doi:10.1158/2767-9764.CRC-22-0336)
Supplement: Fig. S6 — GSEA in LAM [file crc-22-0336-s06.docx]

**
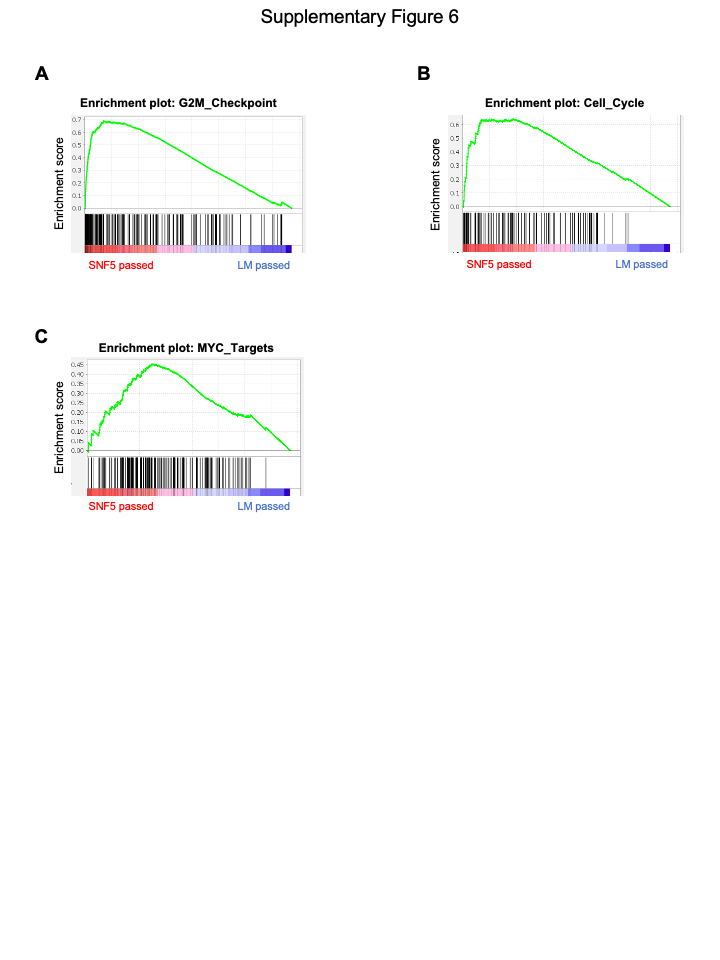
**

**Supplementary Figure 6.**  **Gene set enrichment analysis in GFP+ lymphoma-associated macrophages.** Splenocytes from lymphoma bearing SNF5 ^fl/fl^, CD4-Cre^+^ (SNF5) or littermate control (LM) mice were adoptively transferred into CD68-GFP reporter mice. Upon engraftment, GFP^+^ cells (LM passed, n=3; SNF5 passed, n=5) were sorted, and bulk RNA-seq performed. Gene set enrichment analysis (GSEA) shows enrichment in proliferation and cell-cycle related signatures in GFP+ macrophages obtained from lymphoma-bearing mice.
