# Supplementary material for: Targeting Lymphoma-associated Macrophage Expansion via CSF1R/JAK Inhibition is a Therapeutic Vulnerability in Peripheral T-cell Lymphomas
Source: Cancer Res Commun. 2022 Dec 30;2(12):1727–37. doi: 10.1158/2767-9764.CRC-22-0336 (PMC10035520; doi:10.1158/2767-9764.CRC-22-0336)
Supplement: Fig. S7 — Identification of dual CSF-1R/JAK inhibitors in high-throughput screen [file crc-22-0336-s07.docx]

**
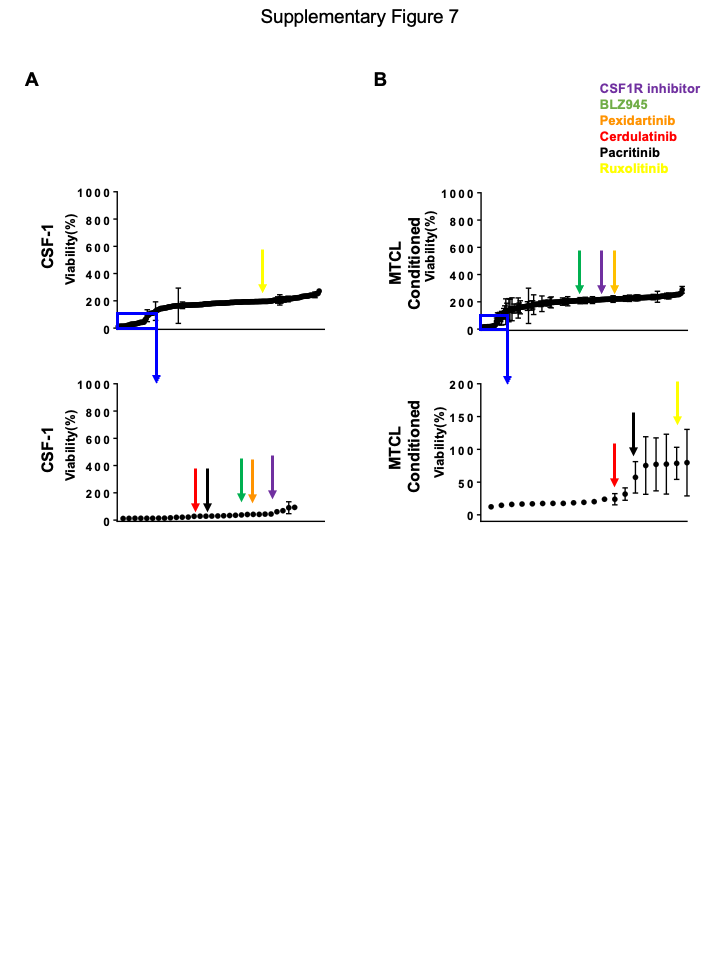
**

**Supplementary Figure 7. A high-throughput screen identifies dual CSF-1R/JAK inhibitors as potent inhibitors of monocyte/macrophage expansion in response to lymphoma-derived CFCM.** Normal donor monocytes were cultured with rhCSF-1 (left panel) or with CFCM (right panel) obtained from a primary TCL specimen. Viability was determined 72 hours later (and normalized to viability at time 0) in triplicate using a 384-well format. Experiments were performed using a library of 191 targeted agents (indicated on x-axis). Cytocidal agents (normalized viability <100%) are shown below. Selected agents, including CSF-1R inhibitors (“CSF-1R inhibitor”, BLZ945, and pexidartinib), ruxolitinib, pacritinib, and cerdulatinib are indicated with the arrows shown.
