# Supplementary material for: Targeting Lymphoma-associated Macrophage Expansion via CSF1R/JAK Inhibition is a Therapeutic Vulnerability in Peripheral T-cell Lymphomas
Source: Cancer Res Commun. 2022 Dec 30;2(12):1727–37. doi: 10.1158/2767-9764.CRC-22-0336 (PMC10035520; doi:10.1158/2767-9764.CRC-22-0336)
Supplement: Table TS1 — Antibodies used [file crc-22-0336-s08.docx]

|  | **Antibodies used for flow cytometry** | | | | |
| --- | --- | --- | --- | --- | --- |
|  | **Antibody** | **Vendor** | **Catalog #** | **Clone** | **Fluorescent** |
| 1 | CD3 | bd biosciences | 562600 | 145-2C11 | BV421 |
| 2 | CD3 |  |  |  | FITC |
| 3 | TCR-Vβ 8.1/8.2 | eBioscience™ | 11-5813-82 | KJ16-133 | FITC |
| 4 | TCR-Vβ 8.3 | bd biosciences | 51-09044L | 1B3.3 | FITC |
| 5 | CD11b | MACS | 130-113-239 | M1/70.15.11.5 | Viogreen |
| 6 | Ly6G | BD Horizon™ | 560603 | 1A8 | V450 |
| 7 | Ly6G | BioLegend | 127605 | 1A8 | FITC |
| 8 | Ly6C | bd biosciences | 553104 | AL-21 | FITC |
| 9 | Ly6C | Invitrogen | 17-5932-82 | HK1.4 | APC |
| 10 | CD193 | BioLegend | 144511 | J073E5 | APC |
| 11 | SiglecF | Invitrogen | 12-1702-80 | 1RNM44N | PE |
| 12 | SiglecF | BioLegend | 155507 | S17007L | APC |
| 13 | CD117 | BioLegend | 105807 | 2B8 | PE |
| 14 | CD16/32 | bd biosciences | 560539 | 2.4G2 | V450 |
| 15 | CD115 | BioLegend | 135509 | AFS98 | APC |
| 16 | FceR1 | BioLegend | 134315 | MAR-1 | APC |
| 17 | CD34 | BioLegend | 152203 | SA376A4 | PE |
| 18 | Ki67 | bd biosciences | 558615 | B56 | APC |
| 19 | CD19 | bd biosciences | 553785 | 1D3 | FITC |
| 20 | NK1.1 | BioLegend | 108705 | PK136 | FITC |
| 21 | F4/80 | Bio-Rad | MCA497R | A3-1 | Purified |
| 22 | MBP | MAB42282 |  | MT-14.7 | Purified |
| 23 | CD8 | Biocare Medical | 3160 | C8/144B | 540 |
| 24 | CD3 | ThermoFisher | MA5-14524 | SP7 | 690 |
| 25 | PLK-1 | Cell Signaling | 4513 | 208G4 | 650 |
| 26 | Ki67 | DAKO/Agilent | M724001-2 | MIB-1 | 620 |
| 27 | CD163 | Abcam | Ab182422 | EPR19518 | 480 |
| 28 | CD68 | ThermoFisher | MA5-12407 | PG-M1 | 570 |
| 29 | CD20 | Leica Biosystems | PA0200 | L26 | 780 |

**Supplementary Table 1. Antibody used for Flwo Cytometry.**
