# Supplementary material for: Targeting Lymphoma-associated Macrophage Expansion via CSF1R/JAK Inhibition is a Therapeutic Vulnerability in Peripheral T-cell Lymphomas
Source: Cancer Res Commun. 2022 Dec 30;2(12):1727–37. doi: 10.1158/2767-9764.CRC-22-0336 (PMC10035520; doi:10.1158/2767-9764.CRC-22-0336)
Supplement: Table TS2 — JAK inhibitor kinase profile [file crc-22-0336-s09.docx]

**Supplementary Table 2. JAK inhibitor kinase profile**

| **“JAK inhibitor” kinase profile (IC_50_, nM)** | | | |
| --- | --- | --- | --- |
|  | **Ruxolitinib {Quintas-Cardama, 2010 #2195}** | **Cerdulatinib {Coffey, 2014 #3942}** | **Pacritinib {Polk, 2016 #3455}{Singer, 2014 #3995}** |
| **JAK1** | 3.3 | 12 | Indeterminate |
| **JAK2** | 2.8 | 6 | 6 |
| **TYK2** | 19 | 0.5 | 27 |
| **JAK3** | 428 | 8 | 18.3 |
| **CSF-1R** | Indeterminate | 5 | 39.5 |
